# Radiological and radioecological risk assessment around the West Delta fossil-fuel power station in Egypt

**DOI:** 10.1038/s41598-025-31092-0

**Published:** 2025-12-27

**Authors:** Shimaa M. Elgingihy, Abdelsalam A. Abdelsalam, Ibrahim H. Saleh

**Affiliations:** 1 Department of Environmental Studies, Institute of Graduate Studies and Research, Alexandria University, Alexandria, Egypt; 2 Department of Soil and Water Sciences, Faculty of Agriculture, Alexandria University, Alexandria, Egypt

**Keywords:** Radioactivity, Environmental risk, Radiological assessment, Radioecological risk, Fossil fuel power plants, Ecology, Ecology, Environmental sciences

## Abstract

There are serious ecological and radiological risks associated with the release and buildup of man-made and natural radionuclides. These risks are particularly relevant for fossil fuel power plants located in residential and agricultural areas. High-purity germanium (HPGe) detectors were employed to analyze environmental samples, including soil, water, and plants collected around the West Delta fossil fuel power station in Egypt. The activity levels of both man-made and naturally occurring radionuclides, such as ^226^Ra, ^228^Ra, and^40^K, were measured, and the corresponding ecological and radiological hazards were assessed using several radiological hazard indices. The findings showed elevated concentrations of ^226^Ra, ^228^Ra, and^40^K specifically in agricultural areas near the power station, with some values exceeding internationally recommended guideline values. The calculated radioecological indicators highlight potential long-term exposure risks for nearby populations and ecosystems. These results indicate the need for targeted monitoring and site-specific mitigation measures in the most impacted areas. while providing essential baseline data for future environmental monitoring. This study provides the first comprehensive radiological and radioecological assessment around the West Delta power station, offering new baseline data for environmental monitoring and risk management.

## Introduction

Fossil fuel power plants have expanded significantly in response to the growing global demand for electricity. These plants frequently release pollutants such as radionuclides, which can accumulate in the surrounding environment. This study investigates the dispersion of radioactive contaminants in residential and agricultural areas near several Egyptian power stations. The objective is to measure the related risks and suggest practical ways to mitigate them^1^.

When rocks and sediments come into contact with water, these radionuclides can dissolve into groundwater. The largest source of natural radioactivity in the Earth’s crust is naturally occurring radioactive materials (NORM), primarily the decay products of ^238^U and ^232^Th, and their buildup in soils or sediments can present serious radioactive hazards to the environment and human health^2^.

Elevated radioactivity and radionuclide accumulation pose significant risks to human and environmental health^3^.

Manufacturing processes involving NORM may present radioactive concerns; thus, it is critical to identify, analyze, and control any potential risks^4^.

By filtering, buffering, and storing materials, soil serves as a geochemical sink for pollutants and is essential to preserving ecosystem equilibrium. Agricultural soils in Egypt’s Northeastern Nile Valley have been shown to serve as a reservoir and distributor of radionuclides and trace elements^5^.

Global attention has increasingly focused on the environmental and health impacts of energy generation. Fossil fuels are among the least expensive energy sources, particularly for developing nations; however, they also have some of the most detrimental effects on the environment^6^.

During the combustion of fossil fuels, naturally occurring radionuclides such as uranium, thorium, and their decay products become concentrated in combustion by-products. These radionuclides are subsequently released into the environment through fly ash emissions, bottom ash disposal, and wastewater discharges, leading to Technologically Enhanced Naturally Occurring Radioactive Materials (TENORM) contamination.

The massive usage of finite fossil fuels like coal, gas, and oil for energy production is causing greenhouse gas emissions to rise at an alarming rate. In order to lower these emissions, scientists are attempting to put into practice, efficient mitigation techniques, such as the usage of renewable energy sources^7^.

Despite extensive international research on the environmental and health hazards of NORM, TENORM, and fossil fuel emissions, studies addressing the radiological and radioecological impacts of fossil fuel power plants in Egypt remain scarce. The West Delta Power Station in Kafr El-Dawar, El-Beheira, in particular, is a crucial location because of its contribution to local energy production and its impact on the ecosystem. This study aims to fill this knowledge gap by conducting a comprehensive radiological and radioecological risk assessment around the West Delta Power Station, thereby providing valuable insights for public health and environmental protection.

## Materials and methods

External terrestrial radiation doses are primarily due to gamma emissions from natural radionuclides, while beta radiation contributes minimally because of its limited penetration in air. ^40^K and the decay products of ^238^U and ^228^Ra (as representative of the Th-232 series) are the main sources of gamma radiation. Rocks, soil, and other earth resources frequently contain these radionuclides.

A gamma-ray spectroscopy system equipped with an HPGe detector was employed to measure the specific activity of radionuclides in samples collected from the Beheira Governorate, particularly in the vicinity of the West Delta Power Station in Kafr El-Dawar. Our study focused on eight specific locations. We also examined the samples for soil texture, heavy metals, and organic matter.

### Study area

#### Historical overview of the station

The Kafr El-Dawar power station is situated on the Mahmoudia Canal in the Qazaz Factory area, approximately 7 km from Kafr El-Dawar city and around 2 km from the agricultural road. The station was established in 1979 with the launch of its first and second units. It was later expanded to include a third unit in 1985 and a fourth unit in 1986.

The station comprises four units, each with a capacity of 110 MW, for a total nominal capacity of 440 MW. It operates on a mixed-fuel system of natural gas and diesel, depending on operational requirements. The water source for the station is the Mahmoudia Canal. The total area of the station is 67 acres, 7 karats, and 19 shares (West Delta Electricity Production Company).

#### Study area description

**Fig. 1:**
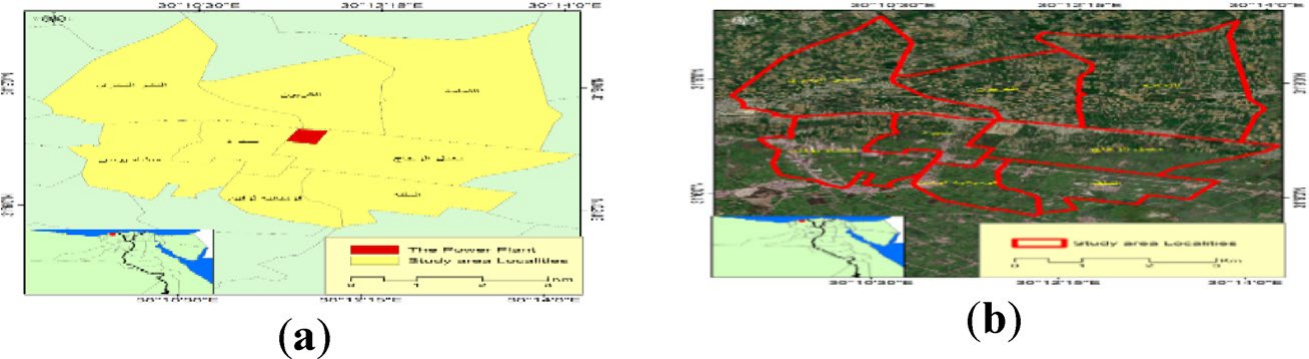
Map of the study area: (**a**) map of sites in the study area; (**b**) map of the study area showing the eight sites.

Comparative radiological environmental research was conducted in eight different areas of the El Beheira Governorate of Egypt, as shown in Fig. 1a : El-Tamama, El-Karyoun, El-Nashw El-Bahary, Maamal El-Zogag, Seira, Manshat Younis, El-Malaqa, and Qombania Loqeen, while Fig. 1b illustrates the locations in detail. The control sample was taken from the Bardala area, which lies outside the research region.

### Experimental model design

A total of forty-one soil samples were collected, represented by the symbol (S) and assigned to Soil Group (A). Six samples were also taken from plants that were growing close to the station; these were assigned to Plant Group (B) and represented by the letter (P). Twelve water samples, designated Water Group (C) and labeled (W), were taken from the neighboring Al-Mahmoudiah Canal. Lastly, as indicated in Table 1, five samples of agricultural wastewater were collected from the vicinity of the station and classified as Wastewater Agriculture Group (D), denoted by the symbol (Ww). The geographic locations of all sampling points were identified using Geographic Information Systems (GIS).

**Table 1:** Experimental model design.

Groups	Samples	Sample Code
(A) Soil group	41	SC, S1, S2, S3, S4, S5, S6, S7, S8, S9, S10, S11, S12, S13, S14, S15, S16, S17, S18, S19, S20, S21, S22, S23, S24, S25, S26, S27, S28, S29, S30, S31, S32, S33, S34, S35, S36, S37, S38, S39, S40
(B) Plant group	6	P1, P2, P3, P4, P5, P6
(C) Water group	12	WC Before, WC After, W1, W2, W3, W4, W5, W6, W7, W8, W9, W10.
(D) Agricultural wastewater group	5	Ww1, Ww2, Ww3, Ww4, Ww5

Where WC Before (control sample taken from a site in Mahmoudia Canal before the study area), and WC After (control sample taken from a site in Mahmoudia Canal after the study area).

Permission for soil and plant sampling was formally obtained from the West Delta Electricity Production Company, as well as from the local agricultural authorities in El-Beheira Governorate. All plant samples were collected exclusively from non-endangered agricultural species.

### Sample collection and preparation

**Fig. 2:**
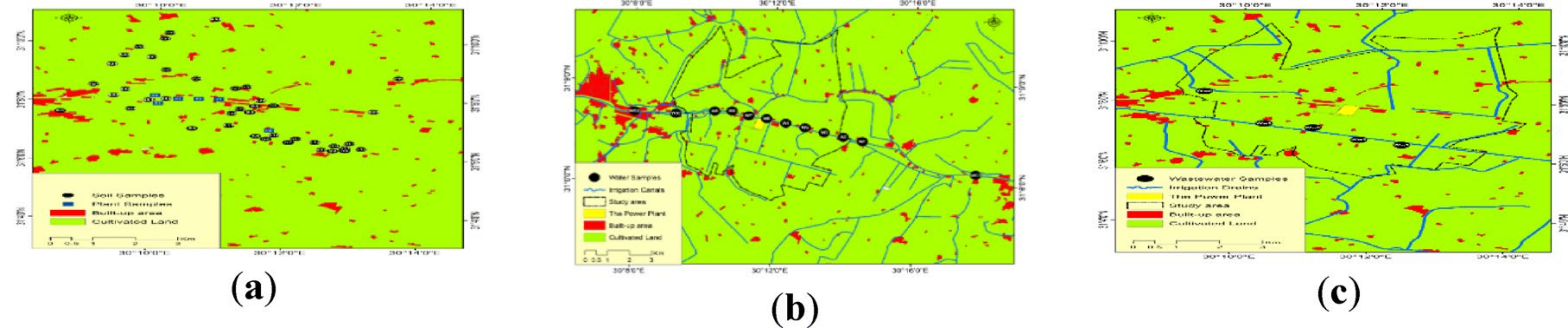
Spatial distribution maps of sampling sites in the study area: **a** soil and plant sampling locations; **b** water sampling locations; **c** agricultural wastewater sampling locations.

Eight towns provided soil samples for the study: El-Malaqa (9 samples), Qombania Loqeen (4 samples), Seira (3 samples), Manshat Younis (4 samples), Nashw El-Bahary (11 samples), El-Karyoun (4 samples), El-Tamama (1 sample), and Maamal El-Zogag (3 samples). In addition, one external control soil sample was collected from Bardalah village outside the influence of the study area. In total, 40 soil samples from the study area and one control sample were obtained Fig. 2a, b, c.

Regarding water sampling, A total of twelve water samples (1 L each) were collected, comprising ten samples from the Al-Mahmoudiya Canal within the study area and two control samples from outside the study area for comparison. Moreover, five agricultural drainage water samples (1 L each) were obtained from sites adjacent to cultivated fields in the study area.

The prepared and collected soil samples were analyzed using a gamma-ray spectrometer equipped with an HPGe detector. The soil samples were also subjected to additional measurements. The coordinates of the research area sites were determined using Geographic Information System (GIS) technology, as shown in Table 2.

**Table 2:** Coordinates of plant samples group (B).

Samples	P1	P2	P3	P4	P5	P6
Scientific name	*Ricinus communis*	*Triticum aestivum*	*Medicago sativa*	*Ficus* *carica*	*Helianthus annuus*	*Artemisia* sp
Plant pictures						

### Soil samples

**Fig. 3:**
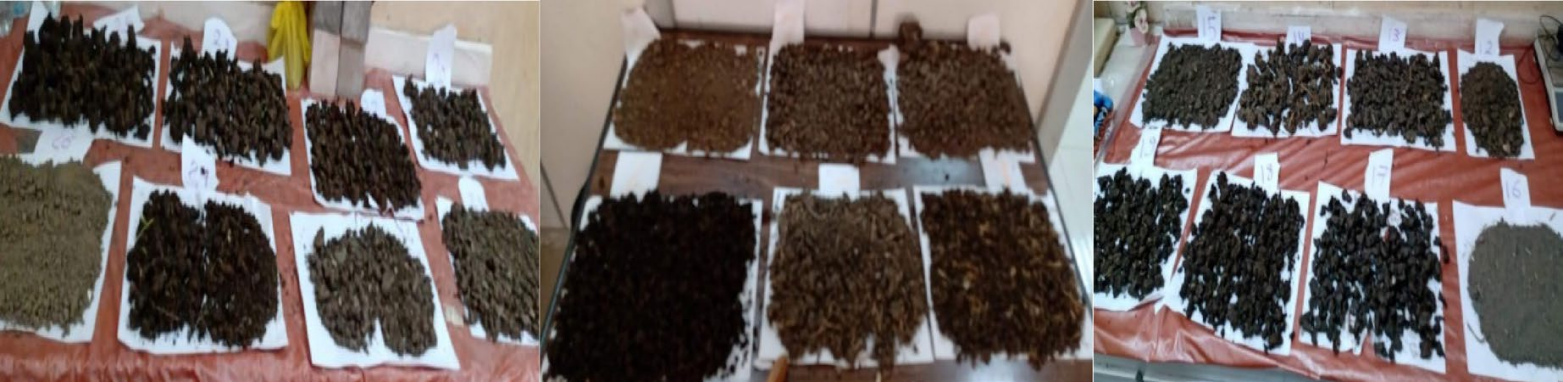
Preparation of soil samples.

Samples of soil (2–3 kg, 5–20 cm depth) were taken at random from agricultural locations (Fig. 3). Following the method outlined by^8^, the samples were ground up, sieved through a 2-mm screen, and dried at 110 °C for 24 h after any visible roots and debris had been removed. To stop radon and thoron from escaping, around 1 kg of the dried material was sealed in plastic containers. Before being analyzed using gamma-ray spectrometry, the sealed samples were allowed to stand for a month to establish secular equilibrium between radium and its short-lived progeny.

These prepared soil samples were subsequently analyzed for radionuclide activity, soil texture, and organic matter content, providing a comprehensive assessment of their environmental characteristics.

### Gamma spectrometry system

The high-purity coaxial detector of the gamma spectroscopy system is composed of pure germanium, a semiconductor that shares properties with silicon. This technique was chosen due to its potent, nondestructive, and selective properties.

The system analyzes a variety of radionuclides present in environmental samples using a 4096-channel multichannel analyzer.

Following gamma spectrometry, the soil samples were further characterized for their texture, organic matter content, and heavy metal concentrations to provide a complete environmental assessment.

Measurement rationale: In this study, ^228^Ra was selected to represent the ^232^Th decay series, as its characteristic gamma emissions can be detected with higher precision and reliability in environmental matrices using HPGe gamma-spectrometry.

The HPGe detector used in this study had a relative efficiency of 18–20% and an energy resolution of 1.8–2.2 keV at the 1.33 MeV gamma line of ^60^Co. The detector was coupled to a low-noise preamplifier, a high-voltage supply (1500–5000 V), and a 4096-channel multichannel analyzer (MCA) operated under a fully computerized system. To minimize background radiation, the detector was shielded by a graded lead assembly lined with cadmium and copper, allowing the accommodation of Marinelli beakers up to 1 L in volume.

Prior to measurements, the system was calibrated for both energy and efficiency using certified reference standards following IAEA guidelines (Moser, 1989). Each soil sample was counted for 80,000 s (≈ 22 h) to achieve high statistical accuracy. Net spectra were obtained by subtracting background counts, and the activity concentrations of ^40^K, ^226^Ra, and ^222^Th were determined from their characteristic gamma lines according to standard procedures (Othman et al. 2018). The overall measurement uncertainty for activity concentration was estimated to be within ± 5%.

### Soil texture analysis in selected soil samples

**Fig. 4:**
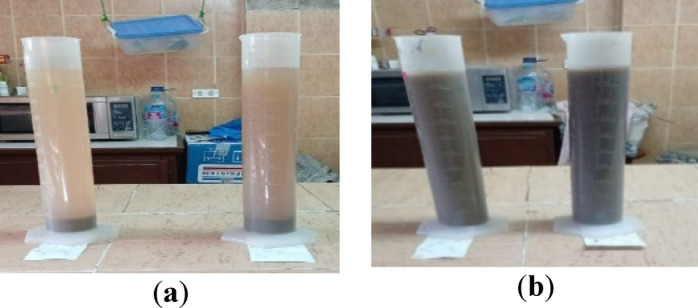
Soil textural classification of the studied samples: (**a**) hydrometer cylinders showing soil suspension for a representative soil sample; (**b**) hydrometer cylinders showing soil suspension for another representative soil sample during the hydrometer test.

Using sodium hexametaphosphate as a dispersing agent, the hydrometer method was used to estimate the soil particle size distribution (PSD), in accordance with^9^. The USDA textural triangle was used to identify soil textural classes based on the measured quantities of sand, silt, and clay Fig. 4a and b.

### Organic matter content (measure) in selected soil samples

**Fig. 5:**
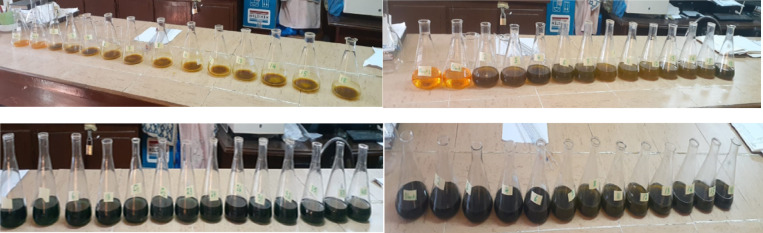
Organic matter analysis.

The traditional Walkley–Black wet oxidation method was used to quantify the amount of soil organic matter (OM)^10,11^. In this process, potassium dichromate (K_2_Cr_2_O_7_) oxidizes organic carbon in the presence of strong sulfuric acid, and ferrous sulfate is used to back-titrate the excess dichromate. Based on the measured organic carbon values, the OM content was computed and presented as a percentage (Fig. 5).

### Heavy metals in selected soil samples

**Fig. 6:**
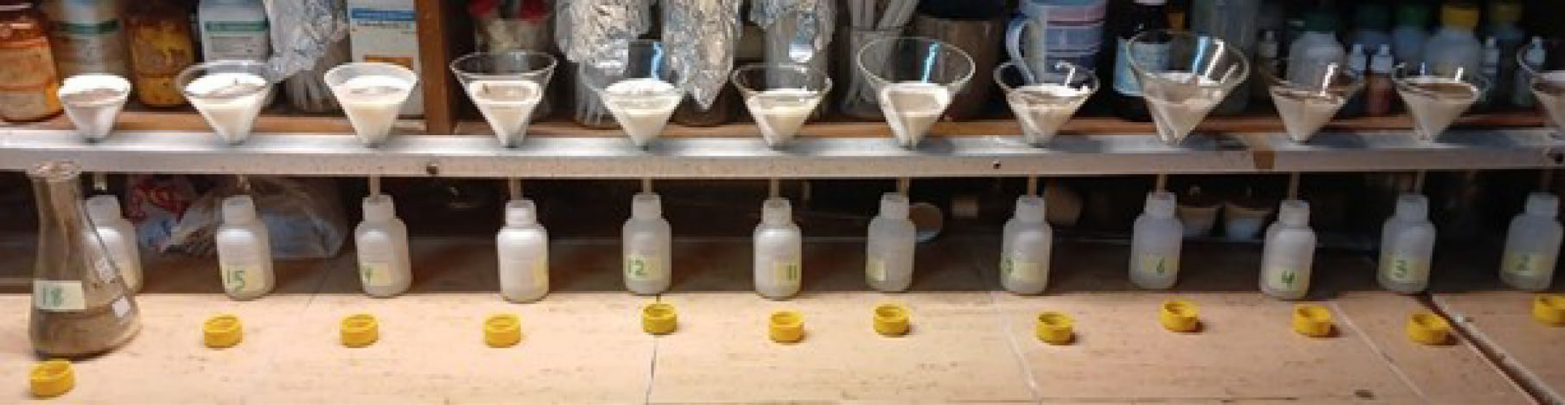
Soil samples after chemical extraction.

Samples of soil were sieved to a particle size of less than 2 mm after being allowed to air dry at ambient temperature. The DTPA method was used to extract the available trace elements (As, Cd, and Pb)^12^. In brief, 10 g of soil was agitated with a DTPA extraction solution for 2 h, an overview of the samples after extraction is shown in Fig. 6. The resulting extracts were subsequently analyzed using ICP-OES to detect trace metals.

#### Inductively coupled plasma—optical emission spectroscopy (ICP-OES- 5100 DVD)

**Fig. 7:**
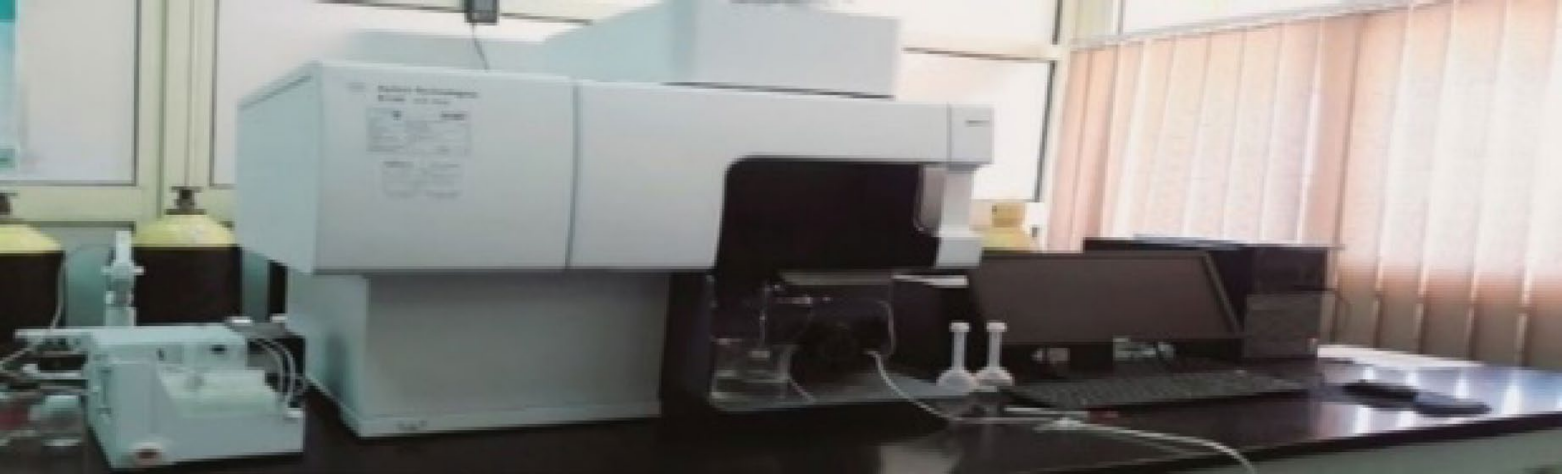
Inductively coupled plasma optical emission spectroscopy (ICP-OES) using Agilent 5100 VDV.

ICP-OES (Agilent 5110, USA) was used to quantify trace elements in soil and water. In accordance with standard procedures, samples were acid-digested before examination (USEPA, 1996)^13^. Multi-element standards were used for calibration, while blanks and approv^−1^ed reference materials were used for QA/QC verification. The findings are expressed as mg L (water) and mg kg^−1^ (soil) (Fig. 7).

Instead of total metal digestion, the diethylenetriaminepentaacetic acid (DTPA) extraction method was employed to determine the bioavailable fractions of trace metals. This approach provides a more accurate estimation of the environmentally mobile and plant-accessible forms of metals rather than their total concentrations, which aligns with the study’s radioecological assessment objectives.

## Results and discussion

### Soil results and discussion

In the soil samples collected, the activity concentrations of both man-made and natural radionuclides were measured. These included the primordial radioactive^40^K, the manmade radionuclides ^137^Cs and^7^Be, and ^226^Ra and ^228^Ra (members of the ^238^U and ^232^Th decay families).

**Fig. 8:**
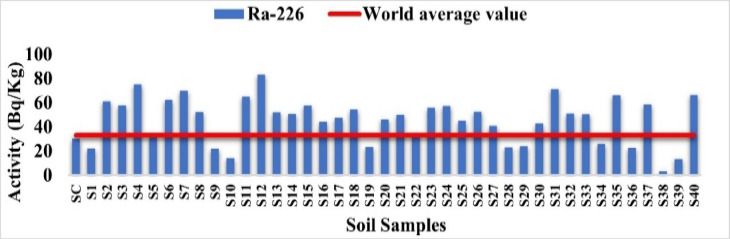
The activity concentration of ^226^Ra in the soil samples collected from the study area (Bq/Kg).

The mean concentration of ^226^Ra was 46.69 Bq/kg, which is higher than the internationally world average value of 33 Bq/kg^14^. The concentrations ranged from 13.55 to 83.27 Bq/kg, with nearly half of the specimens above the reference value, suggesting somewhat elevated levels of uranium-series radionuclides in some areas, whereas Fig. 8 illustrates the activity concentration of ^226^Ra.

^228^Ra was intentionally selected to represent the ^232^Th decay series because it is directly measurable by gamma spectrometry and provides a reliable indication of thorium-series activity in environmental samples.

**Fig. 9:**
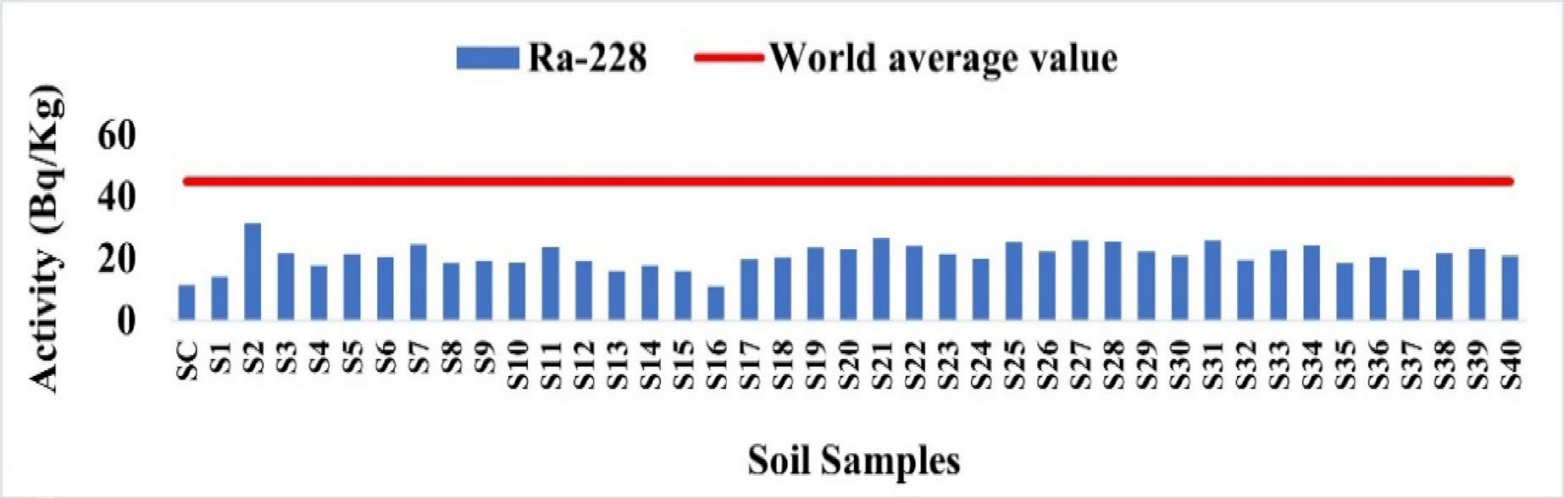
The activity concentration of ^228^Ra in the soil samples collected from the study area (Bq/Kg).

The average concentration of ^228^Ra was 20.95 Bq/kg, with a range of 11.20 to 31.34 Bq/kg. This is significantly less than the standard level of 45 Bq/kg set by world average value^15^. These findings, as shown in Fig. 9, indicate a decrease in thorium activity throughout the research sites.

**Fig. 10:**
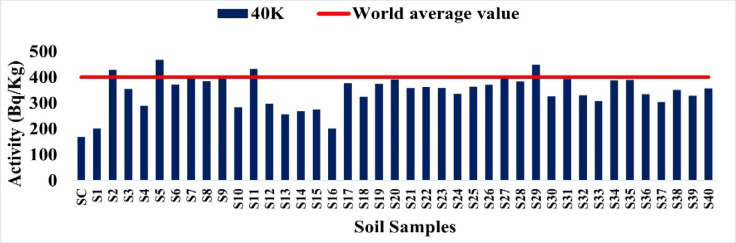
The activity concentration of ^40^K in the soil samples collected from the study area (Bq/Kg).

With an average of 345.33 Bq/kg, the^40^K activity ranged from 169.3 Bq/kg to 467.2 Bq/kg. As seen in Fig. 10, the majority of samples fell below the 400 Bq/kg world average value; however, some (such as S18 and S10) were somewhat above it. These values fall within the typical ranges reported for agricultural and natural soils worldwide.

**Fig. 11:**
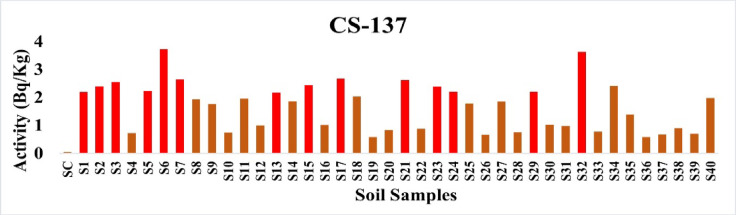
The activity concentration of ^137^Cs in the soil samples collected from the study area (Bq/Kg).

Low levels of anthropogenic ^137^Cs were found, with an average of 1.66 Bq/kg and a range of 0.05 to 3.73 Bq/kg. The measured amounts of this radionuclide in soil are within known background levels from worldwide air fallout events, despite the fact that there is no regulatory limit for it^16^. Figure 11 shows a visualization of the data.

**Fig. 12:**
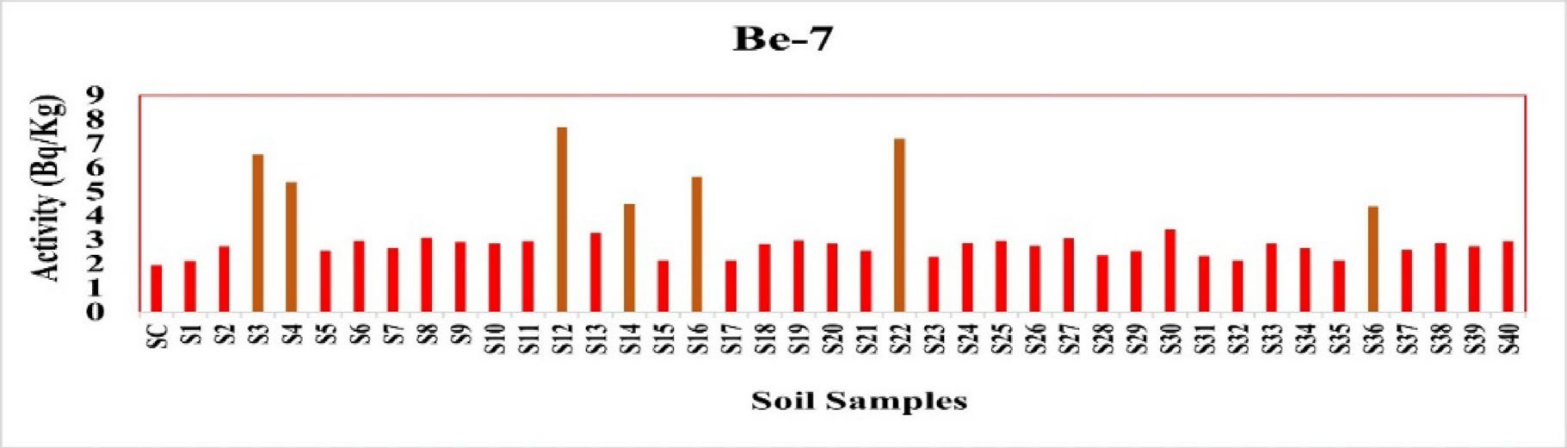
The activity concentration of ^7^Be in the soil samples collected from the study area (Bq/Kg).

The mean concentration of ^7^Be, a short-lived cosmogenic radionuclide, was 3.23 Bq/kg, with a range of 1.96 to 7.69 Bq/kg. Because^7^Be has a dynamic atmospheric origin, there is no reference limit for it in soils; nonetheless, the measured quantities are in line with typical surface deposition from recent rainfall or atmospheric activity^17^. These trends are seen in Fig. 12.

The control soil sample (SC) recorded the lowest values for the main radionuclides presented in the figures, particularly ^226^Ra (30.16 Bq/kg) and ^228^Ra (11.47 Bq/kg), along with a low concentration of ^40^K (169.3 Bq/kg) and background levels of ^137^Cs (0.05 Bq/kg). These consistently low concentrations confirm its reliability as a natural baseline for comparison with the other studied soils.

### Th/U ratio in soil samples

One popular geochemical metric for determining the relative mobility and abundance of naturally occurring radionuclides in soils and sediments is the thorium-to-uranium (Th/U) ratio. The Th/U ratios in this work were calculated using the activity concentrations of ^228^Ra (as a proxy for ^232^Th) and ^226^Ra (as a proxy for ^238^U)^18^.

**Fig. 13:**
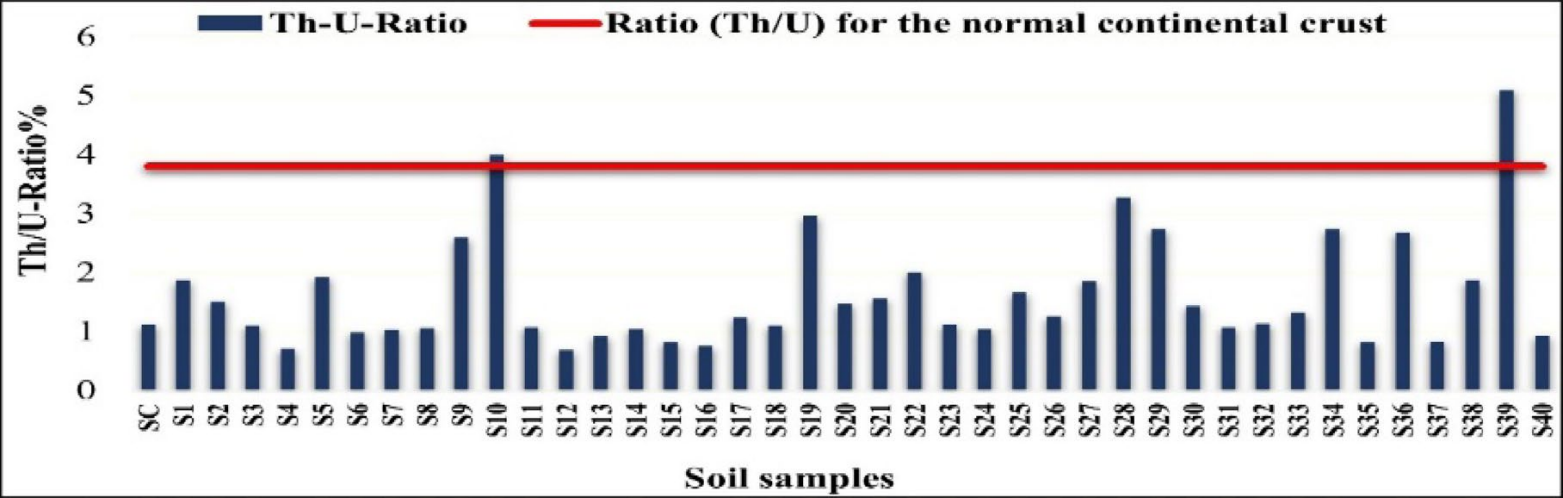
Th/U ratio in soil samples (%).

The Th/U ratios averaged 1.96 (Fig. 13), ranging from 0.00 (S12) to 5.09 (S39). This mean value is lower than the reference upper continental crust ratio of ~ 3.8, indicating relative uranium enrichment. Because thorium is largely immobile and uranium is redox-sensitive and more mobile under oxidizing conditions, low Th/U ratios (observed in 92.5% of the samples) are consistent with expected geochemical behavior^19^.

High Th/U ratios (> 3.8) were detected in about 7.5% of the samples, most notably at site S39 and to a lesser extent S10. These elevated ratios may reflect differential weathering that preferentially mobilizes uranium, or local thorium enrichment associated with resistant accessory minerals such as zircon and monazite. The proximity of several sampling sites to the West Delta fossil-fuel power station also suggests potential anthropogenic inputs, including atmospheric deposition, liquid effluents, or phosphate-based fertilizers, which can disturb the natural Th/U balance. The anomalously high value at site S39 is therefore of particular scientific interest and warrants further mineralogical and geochemical investigation^20,21^.

### Plant results and discussion

The findings show that the activity concentrations of radionuclides varied among the studied plant species (Table 3).

**Table 3:** Coordinates of plant samples group (B).

Radionuclide	Mean (Bq/kg)	Min (Bq/kg)	Max (Bq/kg)	Highest accumulating plant	Lowest accumulating plant
^226^Ra	15.74	0.02	30.27	*Triticum aestivum* (P2)	*Artemisia *sp. (P6)
^228^Ra	9.21	1.14	22.01	*Triticum aestivum* (P2)	*Ricinus communis* (P1)
^40^K	197.07	0.02	383.3	*Helianthus annuus* (P5)	*Artemisia *sp. (P6)
^137^Cs	0.96	0.52	1.37	*Helianthus annuus* (P5)	*Triticum aestivum* (P2)
^7^Be	21.36	0.06	44.64	*Ricinus communis* (P1)	*Artemisia *sp. (P6)

**Fig. 14:**
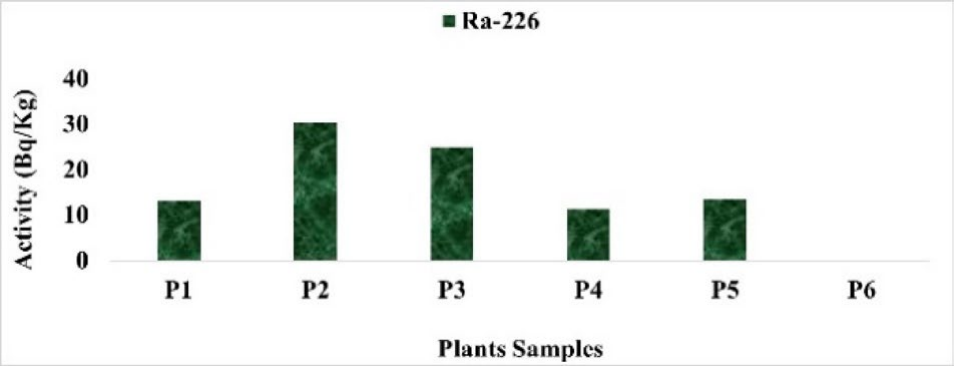
The activity concentration of ^226^Ra in plants collected from the study area (Bq/Kg).

**Fig. 15:**
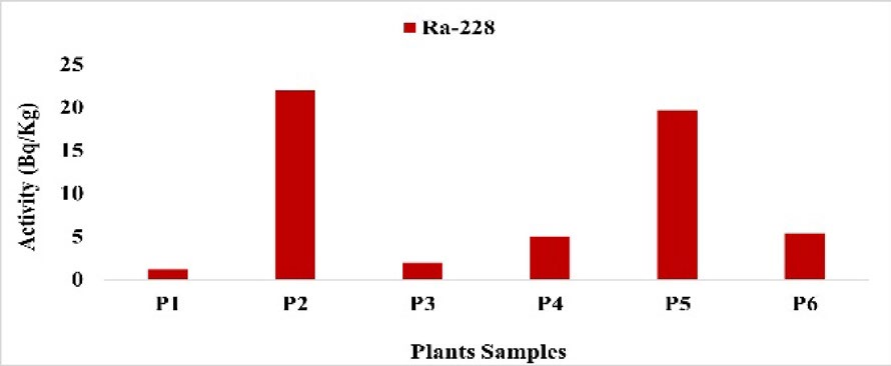
The activity concentration of ^228^Ra in plants collected from the study area (Bq/Kg).

**Fig. 16:**
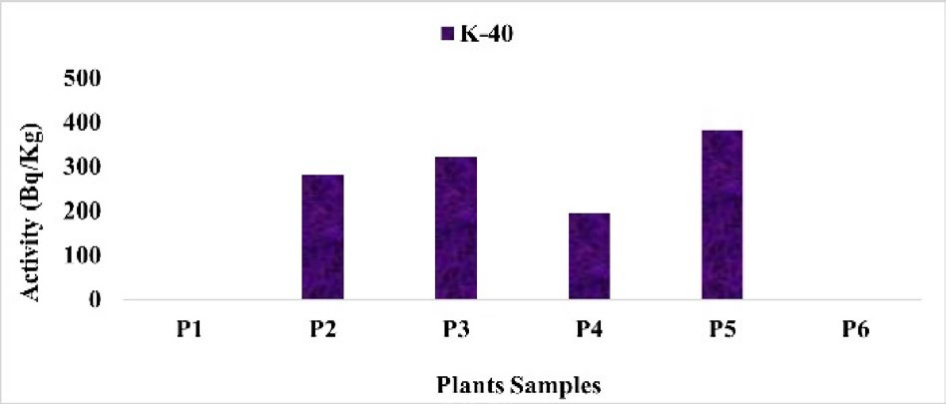
The activity concentration of ^40^K in plants collected from the study area (Bq/Kg).

**Fig. 17:**
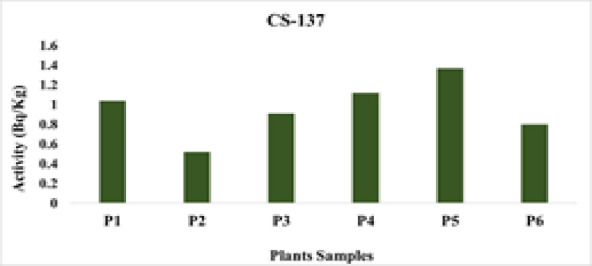
The activity concentration of ^137^Cs in plants collected from the study area (Bq/Kg).

The wide variability in radionuclide activities among the studied plant species reflects differences in radionuclide uptake, likely influenced by both species-specific physiological traits and soil characteristics. Despite some plants exhibiting relatively higher concentrations, the estimated internal dose and lifetime cancer risk remain very low, indicating minimal radiological health concern for human consumption (Figs. 14, 15, 16, 17).

### Transfer factors of radionuclides

**Fig. 18:**
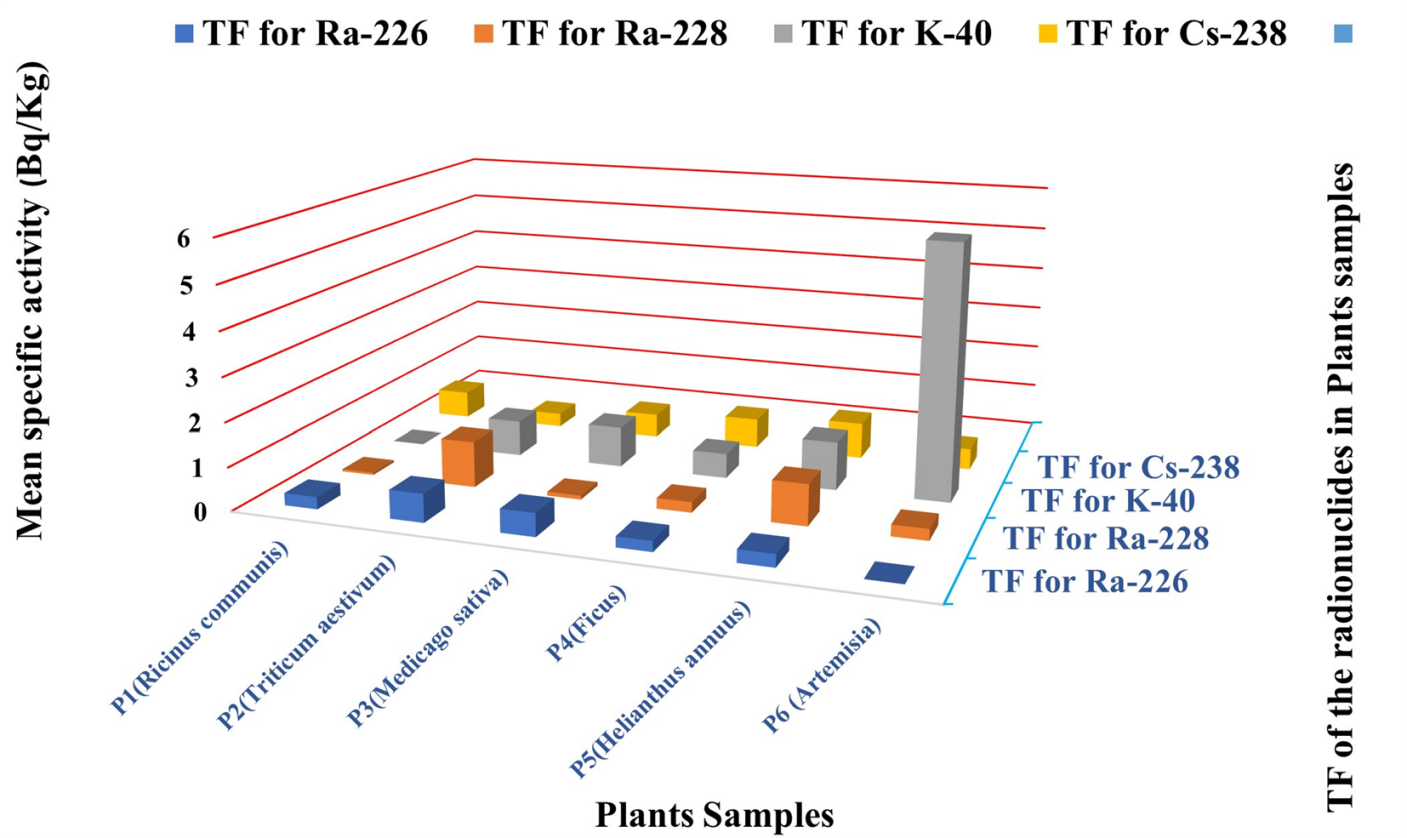
Transfer factor of ^226^Ra, ^228^Ra, ^40^K, and ^137^Cs in plant samples (P1–P6) collected near the power station (Bq/Kg).

**Fig. 19:**
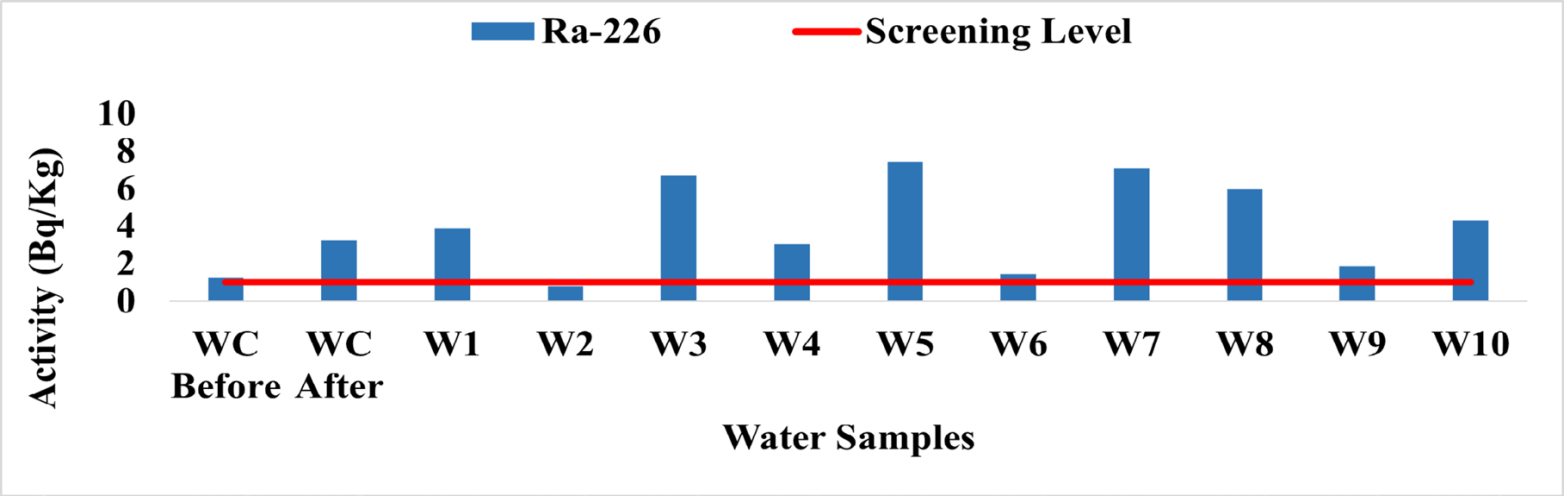
The activity concentration of ^226^Ra in the water samples collected from the study area (Bq/L).

The plant samples collected around the West Delta Kafr El-Dawar power station showed clear interspecific differences in soil-to-plant transfer factors (TFs) for ^226^Ra, ^228^Ra, ^40^K, and ^137^Cs (Fig. 18 and 19).

For ^226^Ra, TFs ranged between 0.28 in *Ricinus communis* (P1) and 0.65 in *Triticum aestivum* (P2).

For ^228^Ra, *Triticum aestivum* also showed the highest TF (1.05), whereas *Ricinus communis* had the lowest (0.05).

For^40^K, *Artemisia* sp. (P6) exhibited the highest TF (5.79), while *Ricinus communis* showed minimal uptake.

For ^137^Cs, TFs varied from 0.32 in *Triticum aestivum* to 0.83 in *Helianthus annuus* (P5).

For^7^Be, high TFs were detected in *Ficus carica* (14.09) and *Ricinus communis* (13.82), whereas *Artemisia* sp. recorded the lowest value (0.02).

The comparatively elevated TFs for^40^K in *Artemisia* sp. and for radium isotopes in *Triticum aestivum* likely reflect differences in root uptake mechanisms and local soil characteristics. Variations among species are consistent with previous studies reporting higher mobility of ^40^K relative to ^226^Ra and ^137^Cs. The elevated^7^Be values may indicate site-specific inputs or short-term deposition processes influenced by local environmental pathways^22^,

These findings underline the need for continued monitoring of radionuclide mobility and plant uptake to better assess potential radiological and radioecological impacts in the study area^23^.

### **Water results and discussion**

The findings show that the activity concentrations of key radionuclides in water samples collected near the West Delta Power Station ranged from 0.81 Bq/L at site W2 to 7.44 Bq/L at site W5 for ^226^Ra, with an average of 3.92 Bq/L. All samples exceeded the WHO preliminary screening guideline of 1 Bq/L. Elevated ^226^Ra levels may result from both natural geological leaching and anthropogenic sources, including fossil fuel combustion and wastewater effluents. As an alpha-emitting nuclide from the uranium-238 decay chain that tends to accumulate in bones, ^226^Ra requires careful consideration in radiological assessments, while the WHO guideline provides an initial safety screening^24^.

**Fig. 20:**
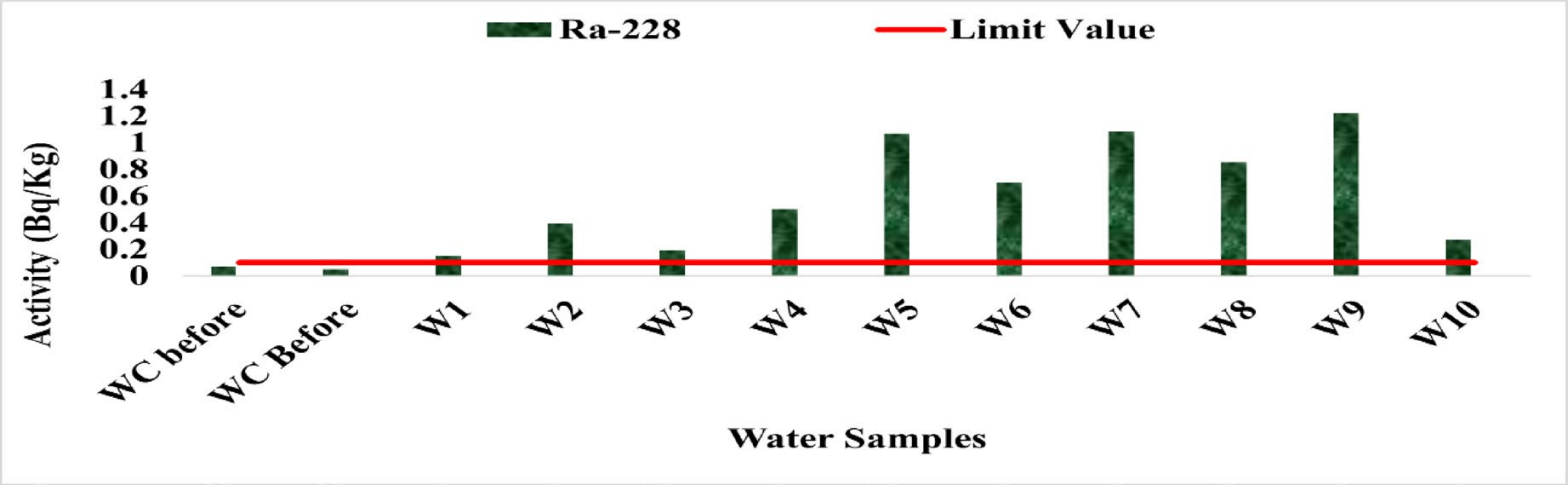
The activity concentration of ^228^Ra in the water samples collected from the study area (Bq/L).

The activity concentration of ^228^Ra ranged from 0.05 Bq/L at site WC After to 1.22 Bq/L at site W9, with an average of 0.55 Bq/L. Figure 20 shows the distribution of ^228^Ra in the water samples, indicating that all studied sites, except the control sample, exceeded the WHO provisional screening level of 0.1 Bq/L for this radionuclide. This clear contrast between the treated control water and the impacted sites highlights the influence of local contamination sources rather than background levels. Elevated ^228^Ra concentrations may originate from thorium-rich geological formations or be enhanced by power station operations, particularly through ash disposal and leachate migration.

**Fig. 21:**
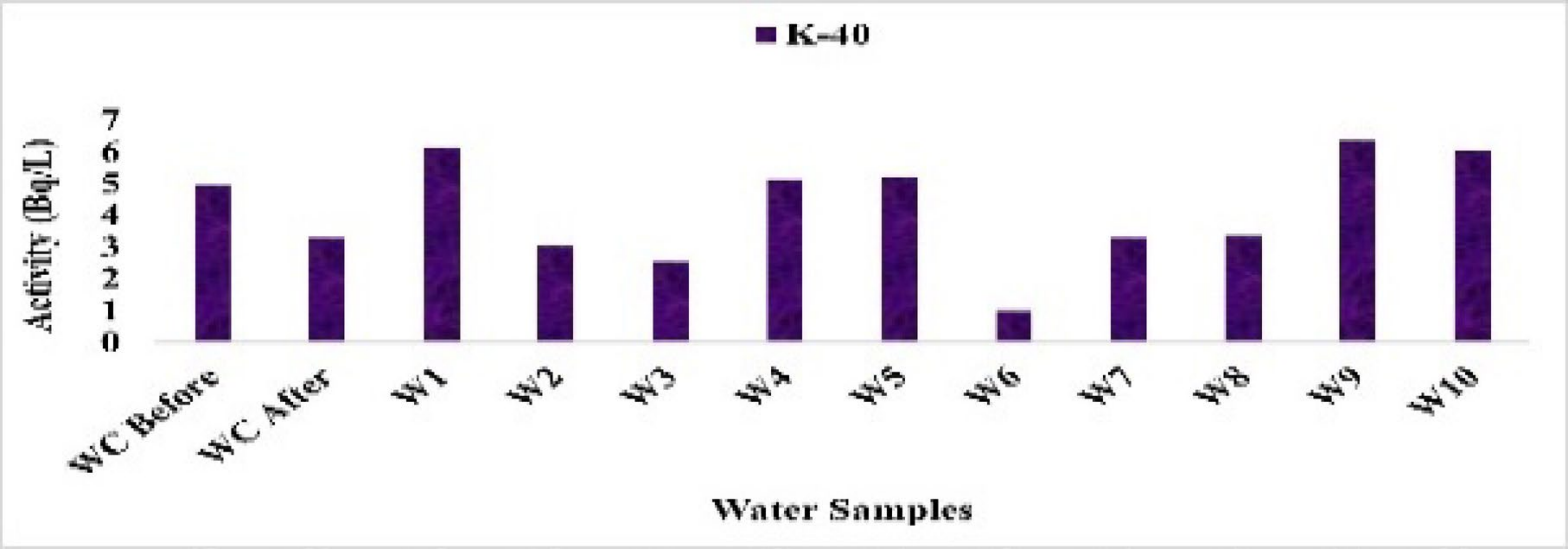
The activity concentration of ^40^K in the water samples collected from the study area (Bq/L).

The activity concentration of 40 K in water samples ranged from 0.99 Bq/L at site WC After to 6.36 Bq/L at site W7, with a mean of 4.19 Bq/L. All measured values were below the WHO provisional guideline value for potassium in drinking water, confirming that the observed concentrations pose no radiological concern, Fig. 21.

**Fig. 22:**
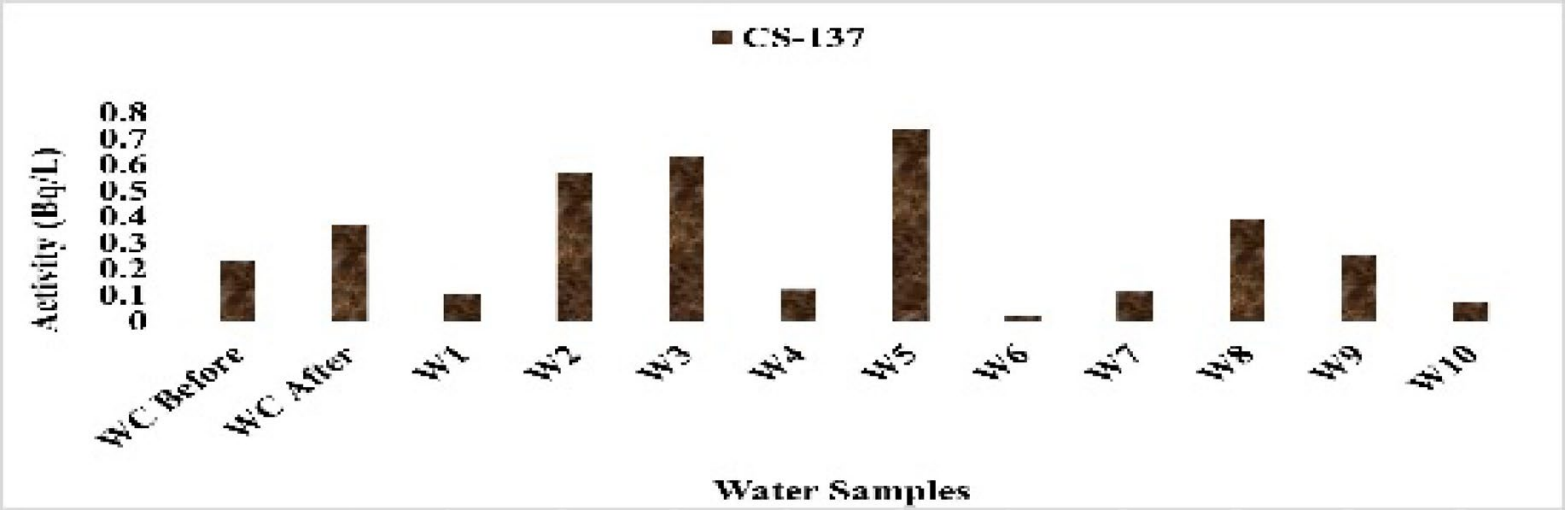
The activity concentration of ^137^Cs in the water samples collected from the study area (Bq/L).

The mean concentration of the anthropogenic radioisotope ^137^Cs was 0.36 Bq/L, with values ranging from 0.06 Bq/L at site WC After to 0.74 Bq/L at site W8. Figure 22 shows the variation of ^137^Cs concentrations among sampling sites. All measured values were below typical global background levels associated with historical atmospheric nuclear testing and fallout events such as the Chernobyl accident. The clear distinction between the treated control sample and elevated concentrations at impacted sites indicates localized anthropogenic inputs. Due to its long half-life and environmental mobility, ^137^Cs requires continuous monitoring, even at low concentrations^25^.

Overall, the control water samples (WC Before and WC After) consistently exhibited the lowest levels of the key radionuclides. ^226^Ra was 1.25 Bq/L in WC Before and 3.25 Bq/L in WC After, compared with higher values such as 6.71 Bq/L at W3. Similarly, ^228^Ra remained low (0.25–0.30 Bq/L) in the controls, while other sites reached up to 0.50 Bq/L. The controls also showed minimal^40^K (4.94 and 3.30 Bq/L) compared with elevated levels in downstream sites, and ^137^Cs stayed close to background (0.23–0.37 Bq/L). These results confirm WC Before and WC After as true background references, against which the radiological enrichment in other canal sites can be clearly assessed.

### Agricultural wastewater results and discussion

Radionuclide concentrations in agricultural wastewater were measured at five sampling sites. The results for ^226^Ra, ^228^Ra, ^40^K, and ^137^Cs are presented below.

**Fig. 23:**
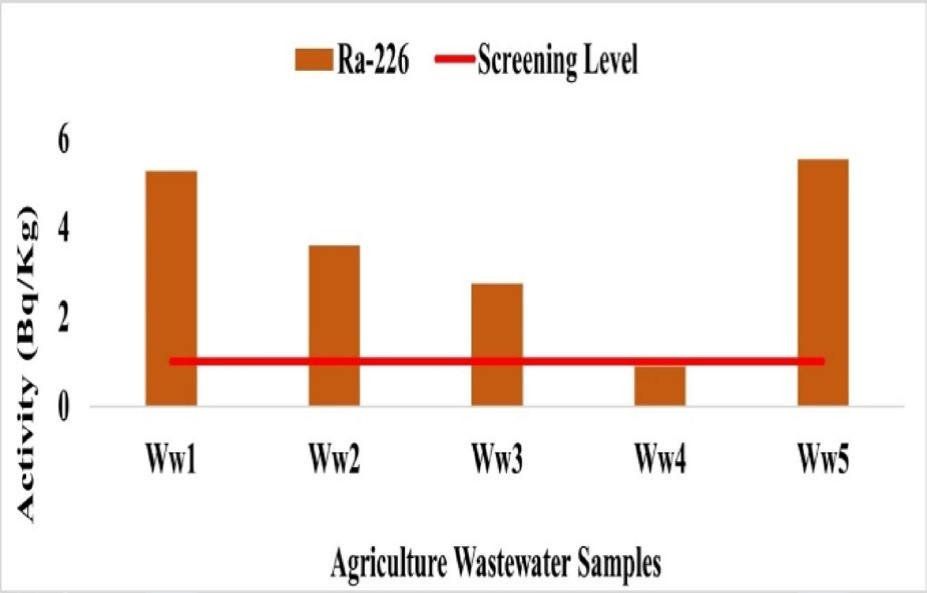
The activity concentration of ^226^Rain the agricultural wastewater samples collected from the study area (Bq/L).

**Fig. 24:**
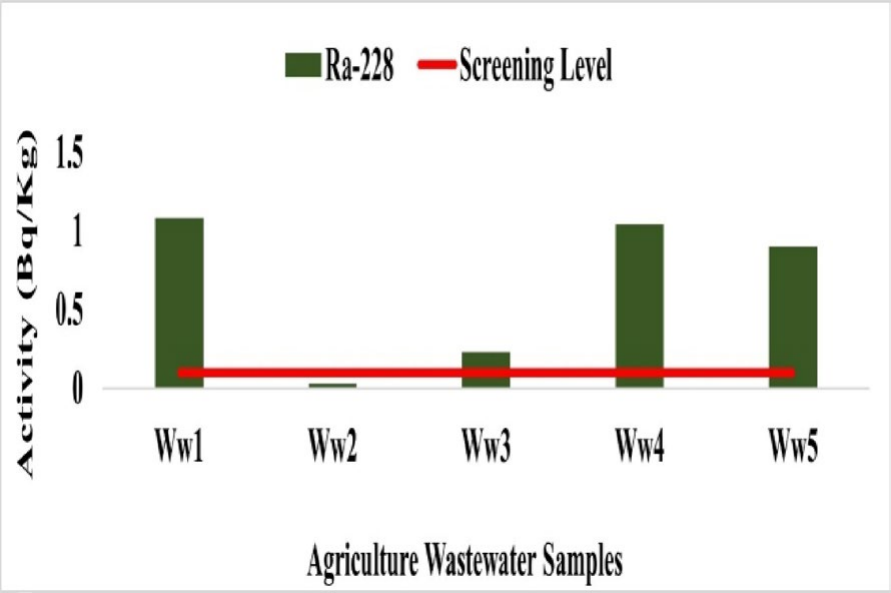
The activity concentration of ^228^Ra in the agricultural wastewater samples collected from the study area.

**Fig. 25:**
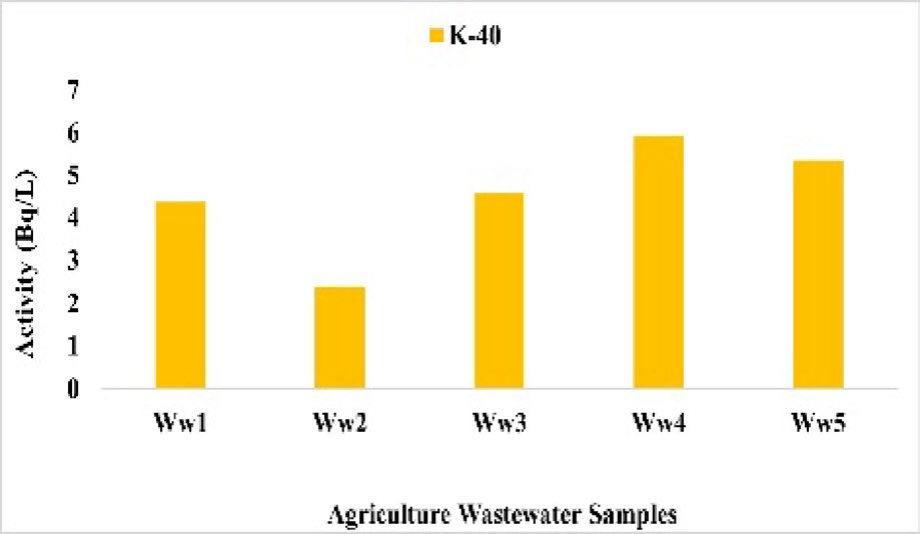
The activity concentration of ^40^K in the agricultural wastewater samples collected from the study area (Bq/L).

**Fig. 26:**
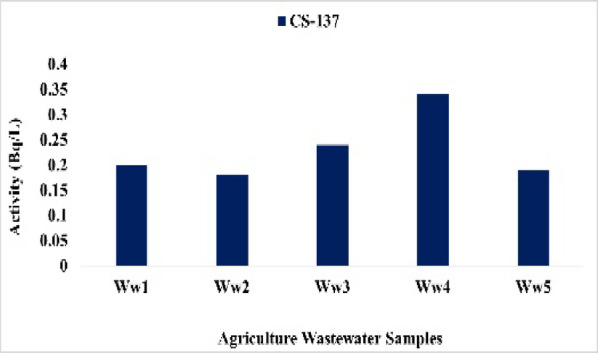
The activity concentration of ^137^Cs in the agricultural wastewater samples collected from the study area (Bq/L).

Radionuclide concentrations in agricultural wastewater ranged as follows: ^226^Ra from 0.89 Bq/L at Ww4 to 5.54 Bq/L at Ww5 with mean 3.61 Bq/L (Fig. 23), ^228^Ra from 0.10 Bq/L at Ww2 to 1.54 Bq/L at Ww5 with mean 1.176 Bq/L (Fig. 24), ^40^K from 2.40 Bq/L at Ww2 to 5.93 Bq/L at Ww1 with mean 4.52 Bq/L (Fig. 25), and ^137^Cs from 0.18 Bq/L at Ww2 to 0.34 Bq/L at Ww4 with mean 0.23 Bq/L (Fig. 26). All ^226^Ra and ^228^Ra values, except at Ww4 (0.89 Bq/L) and Ww2 (0.10 Bq/L), exceeded the WHO preliminary screening levels of 1 Bq/L and 0.1 Bq/L, respectively, whereas^40^K and ^137^Cs remained within typical environmental background levels and below recommended screening limits.

The measured radionuclide concentrations indicate minimal radiological concern overall. Elevated ^226^Ra levels at most sites warrant monitoring, as it is an alpha-emitting radionuclide that can accumulate in bones if ingested, potentially affecting bone tissue. Higher ^228^Ra at selected sites may reflect thorium-series enrichment influenced by local anthropogenic or industrial inputs. ^40^K remained within typical environmental background levels, and ^137^Cs activity was low, confirming negligible contamination from artificial sources.

### **Radioecological assessment**

In order to analyze potential radiological dangers, the radium equivalent activity (Ra _eq_) for the soil samples in order to determine their radioecological status.

#### Radium equivalent activity (Ra _eq_) in soil

To compare the specific activities of samples containing different amounts of ^226^Ra, ^228^Ra and^40^K, the radium equivalent activity (Ra_eq_) was calculated according to the widely used formula OECD^26^:

$${\mathrm{Radium}}\;{\mathrm{equivalent}}\;\left( {{\mathrm{Bq}}/{\mathrm{Kg}}} \right) = {\mathrm{A}}_{{{\mathrm{Ra}}}} + 1.43{\mathrm{A}}_{{{\mathrm{th}}}} + 0.077{\mathrm{A}}_{{\mathrm{K}}}$$

where A_Ra_, A_Th_, A_K_ are the activity concentrations of ^226^Ra, ^228^Ra and^40^K, respectively.

**Fig. 27:**
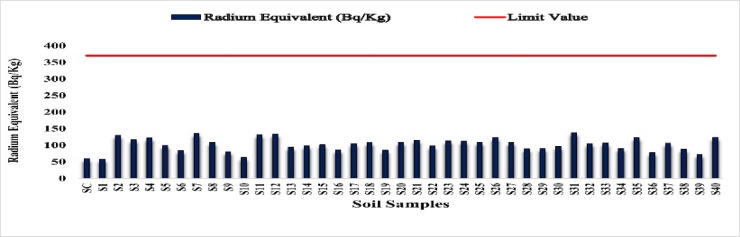
Radium equivalent activity in soil samples (Bq/Kg).

The calculated values of Ra _eq_ for the soil samples are presented in Table 2. Ra _eq_ values ranged between 58.02 Bq/kg at to 138.85 Bq kg^−1^, with a mean value of 103.51 Bq kg^−1^. These values are below the recommended maximum value of 370 Bq kg^−1^ set by the OECD^27,28^. The variation of Ra _eq_ values is shown in Fig. 27, The control site (SC) had one of the lowest Ra _eq_ values, confirming its use as a background reference.

The measured radionuclide activities fall within typical global ranges, with all Ra _eq_ values remaining below the recommended limit of 370 Bq/kg. These results indicate that the study area is radiologically stable, and the observed variations in Ra _eq_ are mainly attributable to natural geological differences in uranium- and thorium-bearing minerals rather than anthropogenic inputs. The absence of industrial discharge, intensive mining, or long-term waste accumulation further supports the minimal influence of human-induced radioactive enrichment in the investigated soils.

### **Radiological assessment**

Soil samples were evaluated for radiological hazards using standard indices, including hazard indices, dose rates, and lifetime cancer and genetic risks. Results and interpretations are presented in the following subsections.

#### External radiation hazard index (H _ex_) of natural radionuclides for soil samples

One of the most important metrics for assessing the radiological risk from gamma radiation in soils is the external hazard index (H_ex_). H_ex_ was calculated using the model of Beretka and Mathew, which ensures that the external gamma dose remains below the recommended annual limit of 1 mSv for the general public. In this study, Hex values ranged from 0.15 to 0.38, with an average of 0.278, well below the allowable maximum of 1. These results indicate that the soils pose no appreciable external radiation risk, supporting their safe use in construction and agriculture.

#### Internal hazard index (H _in_) of natural radionuclides for soil samples

In this study, (H_in_) was calculated using measured activity concentrations of ^226^Ra, ^228^Ra, and ^40^K in soil samples from the West Delta Power Station area. Values ranged from 0.167 to 0.575, with a mean of 0.401, all below the recommended limit of 1^30‚31^. These results indicate that internal exposure from radon and thoron inhalation is low and within accepted radiological safety margins.3.8.3 Radiation-Absorbed Dose Rate for Soil Samples.

#### Radiation-absorbed dose rate for soil samples

The absorbed gamma dose rate (D) in air at 1 m above the ground surface due to the activity concentrations of ^226^Ra, ^232^Th and^40^K was calculated using the following equation recommended by UNSCEAR (2000):

$${\mathrm{D}}\left( {\frac{{{\mathrm{nGy}}}}{{\mathrm{h}}}} \right) = 0.462\;{\mathrm{A}}_{{{\mathrm{Ra}}}} + 0.604{\mathrm{A}}_{{{\mathrm{th}}}} + 0.0417{\mathrm{A}}_{{\mathrm{K}}}$$

**Fig. 28:**
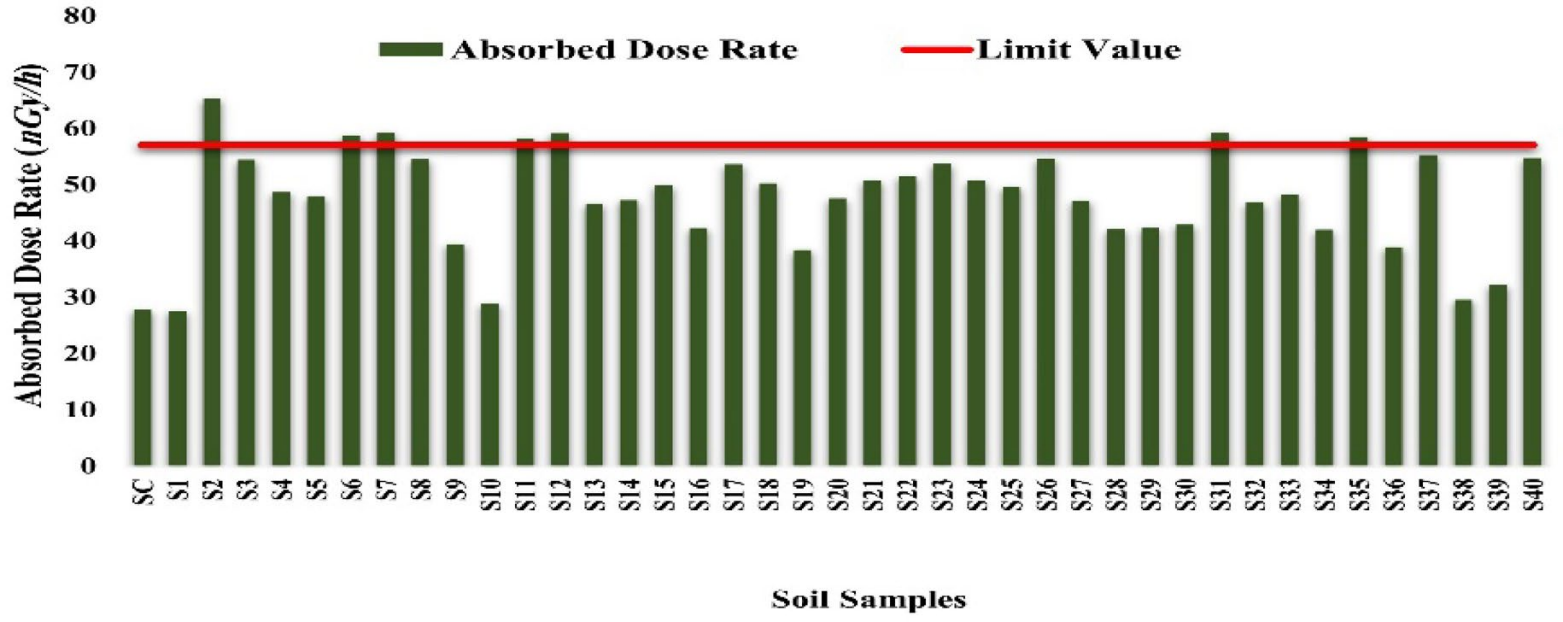
Absorbed dose rate for soil samples (*nGy/h*).

The calculated absorbed gamma dose rate (D) in air at 1 m above ground ranged from 27.54 to 65.33 (nanograys per hour) nGy h^−1^, with an average value of 47.71 nGy h^−1^. These values are below the global average dose rate of 59 nGy h^−1^ for soil^32^, as reported by UNSCEAR (2000). The distribution of the absorbed dose rate is presented in Fig. 28.

Some samples (S2, S3, S6, S7, S11, S12, S17, S37, S40) approached or exceeded 55 nGy/h, showing localized increases in natural radioactivity. These increases are likely due to the accumulation of ^40^K, ^226^Ra, and ^228^Ra, potentially influenced by emissions from the power plant or related industrial activities. Although current levels remain below international safety limits, chronic exposure in residential and agricultural areas could lead to cumulative radiological effects. The spatial distribution of absorbed dose rates supports these observations.

#### The annual effective dose (AED) in soil samples

**Fig. 29:**
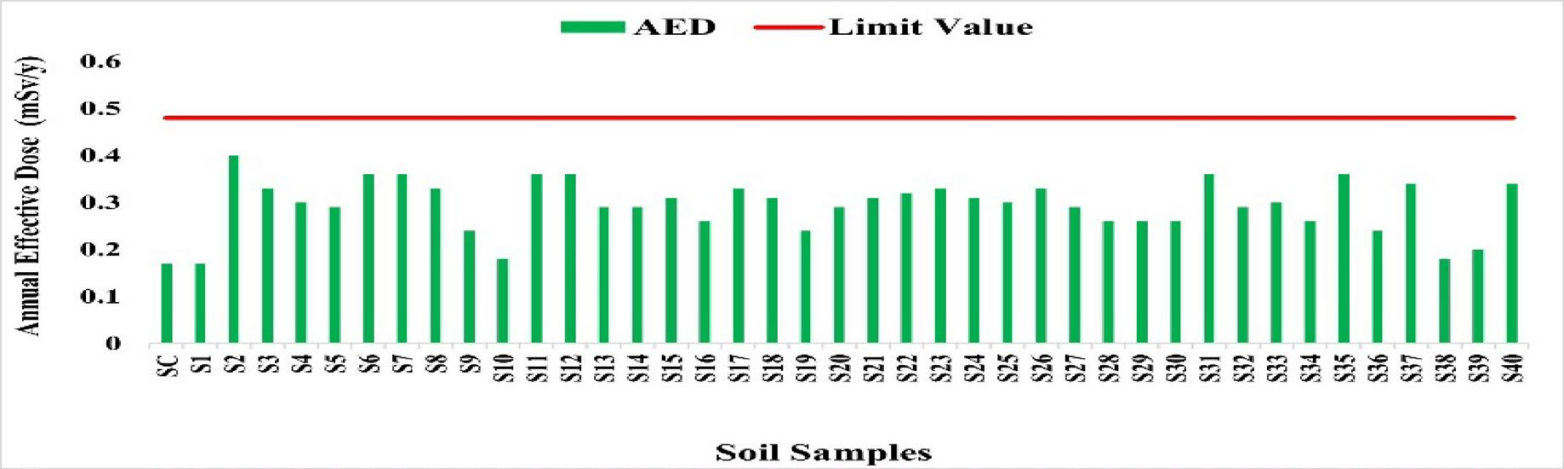
The annual effective dose (AED) for soil samples (mSv/y).

According to Fig. 29, the average annual effective dose (AED) from external gamma radiation in soil samples was 0.292 mSv/y, ranging from 0.17 mSv/y at SC and S1 to 0.40 mSv/y at S2. This is below the global average outdoor dose of 0.48 mSv/y (UNSCEAR)^33^. No substantial radiological risk was observed, although localized increases at S2 warrant further monitoring.

The control site (SC) recorded the lowest radionuclide activities (^226^Ra: 30.16 Bq/kg, ^228^Ra: 11.47 Bq/kg, ^40^K: 169.3 Bq/kg, ^127^Cs: 0.05 Bq/kg) and the minimum absorbed dose rate (27.76 nGy/h) and annual effective dose (0.17 mSv/y) compared with maxima of 65.33 nGy/h and 0.40 mSv/y. These results confirm SC as a reliable background reference essential for evaluating enrichment patterns in the studied soils.

#### Excess lifetime cancer risk (ELCR) in soil samples

The soil samples close to the West Delta Power Station in the current investigation had ELCR values ranging from 0.00011 to 0.00032, with an average of 0.000218. With a mean of 218 incidences per million, these values correspond to an estimated 110 to 320 cancer cases per million people.

This average is still below the US Environmental Protection Agency’s (USEPA) globally advised threshold of 1.0 × 10^−2^. Additionally, according to the United Nations Scientific Committee on the Effects of Atomic Radiation, it is less than the average lifetime cancer risk worldwide, which is 2.9 × 10^−2^. Although routine environmental monitoring is recommended to detect any future radioactive changes due to natural or anthropogenic activities, these data imply that the radiological cancer risk associated with soil exposure in the study area is now within acceptable safety standards.

#### Excess lifetime genetic risk (ELGR) in soil samples

ELGR values were computed for soil samples in this investigation; the mean value was 0.210 × 10^−2^, with a range of 0.119 × 10^−2^ to 0.280 × 10^−2^. With an average of 210 instances per million, these numbers translate to an estimated 119 to 280 possible genetic cases per million people.

A slight but significant genetic risk is indicated by the research area’s mean ELGR, which is higher than the usual worldwide reference value of 0.21 × 10^−2^ when compared to international norms. Even while the numbers are not particularly high, they do highlight the significance of continuous environmental monitoring, public education, and preventative actions, particularly in areas with greater exposure levels.

### The texture of selected soil samples in the study area

Selected soil samples, characterized by relatively higher radioactivity levels, were analyzed to determine their textural properties^34^.

One of the most important factors in soil mechanics, especially in geotechnical and environmental engineering, is particle size. In this approach, particle size distribution (PSD), or soil texture, is crucial. PSD is a useful technique for classifying soil and comprehending its mechanical and hydrological behavior by taking specific gravity, cohesiveness, and permeability into account^35^.

**Fig. 30:**
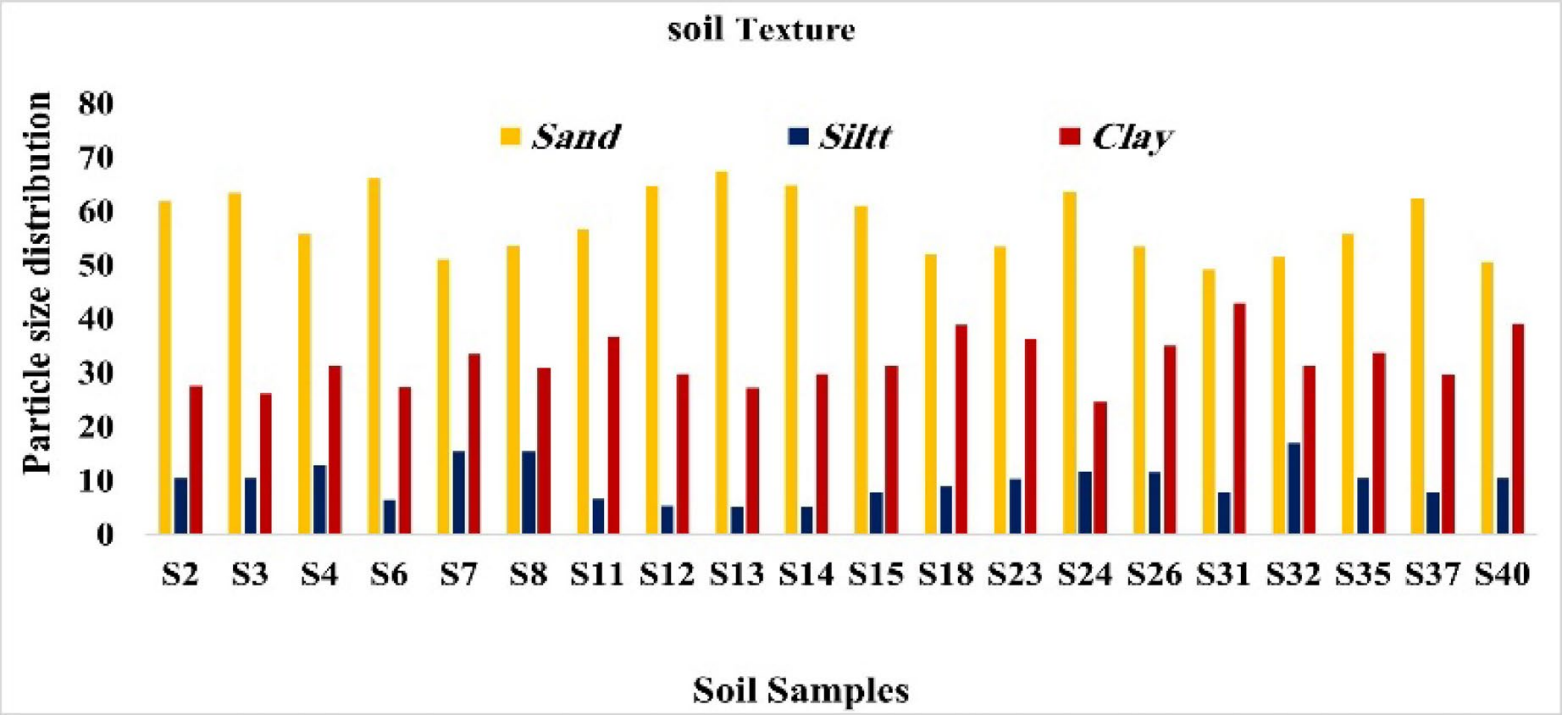
Texture classes of selected soil samples (%).

According to the soil texture study shown in, 30% of the samples were classed as sandy clay, and 70% of the samples were classified as sandy clay loam. At sample position S31, the sand concentration was 49.2%, while at S13, it was 67.5%. At S13 and S14, the silt concentration was 5.2%, but at S32, it was 16.9%. The percentage of clay ranged from 24.7% (S24) to 43% (S31). Shown in Fig. 30.

### Organic matter in selected soil samples

Selected soil samples with relatively elevated radioactivity were analyzed for their organic matter (OM) content. Soil OM, comprising humic substances, microbial biomass, and decomposed plant and animal residues, is influenced by soil type, climate, vegetation, and land management^36^, and plays a critical role in nutrient retention, soil structure, water-holding capacity, and pollutant mobility, including radionuclides. OM can bind radionuclides, forming stable complexes that affect their bioavailability and mobility^37^.

**Fig. 31:**
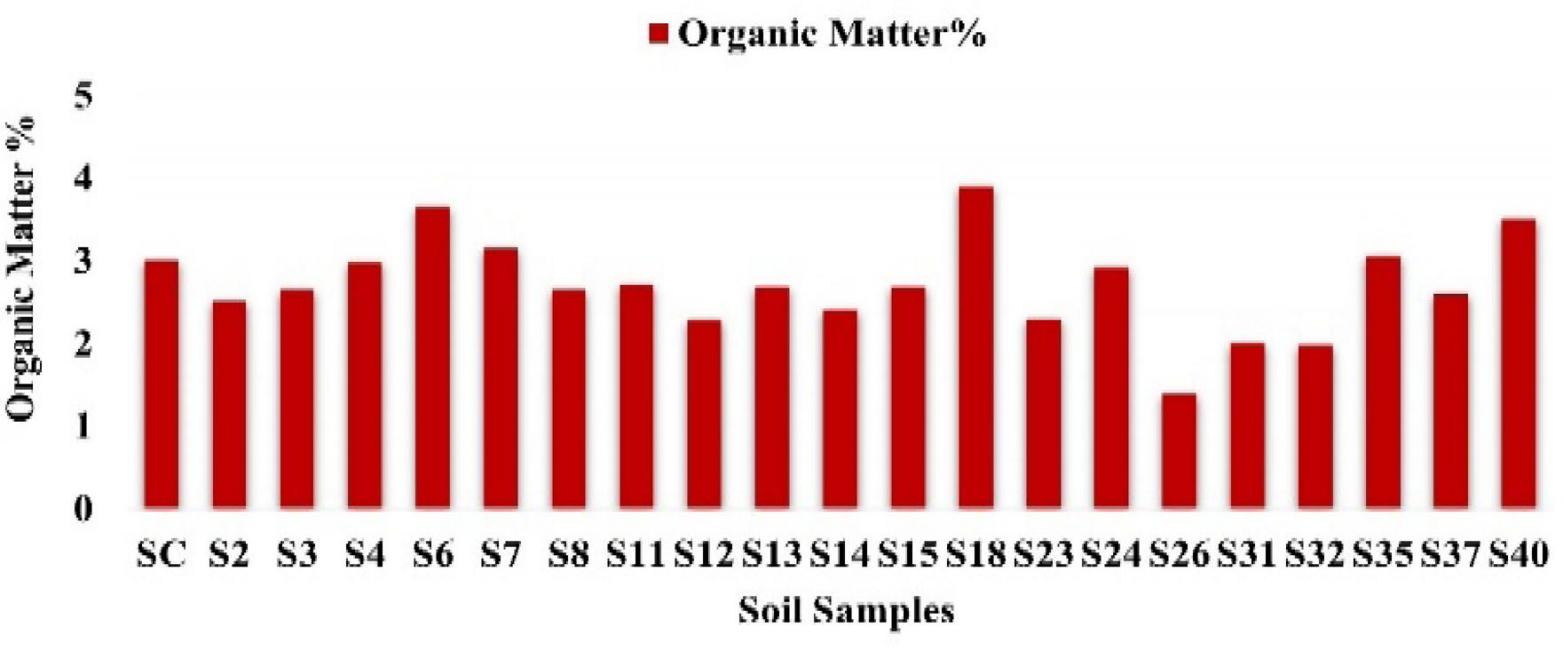
Organic matter content of the soil samples (%).

In this study, 20 soil samples (SC, S2, S3, S4, S6, S7, S8, S11, S12, S13, S14, S15, S18, S23, S24, S26, S31, S32, S35, S37, S40) exhibited OM contents ranging from 1.39% (S26) to 3.90% (S18), with a mean of 2.72% (Fig. 31). According to standard classification, 65% of the samples were mineral soils (< 3% OM), while 35% were mineral soils with organic content (3–15% OM). This distribution highlights the significance of OM in controlling the behavior and fate of radioactive contaminants in soil systems.

### Heavy metal concentrations in selected soil samples

Selected soil samples, identified by their higher radioactivity levels, were analyzed for heavy metal concentrations (Pb, Cd, and As). The amounts of these metals were assessed against international environmental standards. Trace metals such as lead (Pb), arsenic (As), and cadmium (Cd) are considered environmentally relevant due to their toxicity, persistence, and bioaccumulation potential, particularly in agricultural and food-producing areas. Their environmental risk is influenced by chemical form, bioavailability, soil accumulation, and plant uptake, directly affecting crop quality, ecosystem health, and food safety. Consequently, assessing their concentrations and mobility is crucial for evaluating environmental hazards and guiding appropriate land management strategies^38^. The levels of Pb, Cd, and As in selected soil samples are presented in Table 4.

**Table 4:** Heavy metal concentrations in selected soil samples.

Samples	As (mg/kg)	Pb (mg/kg)	Cd (mg/kg)	Samples	As (mg/kg)	Pb (mg/kg)	Cd (mg/kg)
SC	0.001	0.006	0.002	S15	0.001	0.182	0.017
S2	0.006	0.685	0.013	S18	0.001	0.935	0.013
S3	0.001	0.734	0.019	S23	0.004	1.424	0.038
S4	0.009	2.628	0.021	S24	0.001	0.572	0.014
S6	0.008	1.083	0.041	S26	0.006	0.314	0.013
S7	0.004	0.494	0.014	S31	0.001	0.234	0.012
S8	0.001	0.524	0.015	S32	0.006	1.932	0.029
S11	0.005	0.476	0.013	S35	0.007	0.355	0.015
S12	0.003	0.821	0.014	S37	0.001	0.440	0.010
S13	0.001	1.735	0.019	S40	0.003	0.674	0.043
S14	0.002	0.855	0.014				

Table 3 shows the Pb, Cd, and As concentrations in the soil samples. Lead was highest in S4, S13, and S32 (0.006–2.628 mg kg^−1^), Cd in S6, S23, and S40 (0.002–0.043 mg kg^−1^), while As remained low (0.001–0.009 mg kg^−1^), all within international limits.

#### Lead (Pb)

Detected values are below worldwide guideline ranges for agricultural soils (70–100 mg/kg; WHO/FAO)38, although S4, S13, S23, and S32 showed higher contents than the local background (1.4–2.6 mg/kg). This relative enrichment likely reflects anthropogenic inputs from the nearby power plant and traffic or industrial emissions. Spatial variability indicates localized impacts on soil quality, highlighting the need for ongoing monitoring^38^.

#### Cadmium (Cd)

Most samples were below the international guideline (0.02 mg/kg; USEPA ^39^, except S23 (0.038 mg/kg). Due to Cd’s high mobility and bioavailability, even small exceedances require attention to prevent accumulation in edible plants39.

#### Arsenic (As)

All values were below the WHO permissible limit of 0.01 mg/kg40. Despite low concentrations, continued monitoring is recommended due to As’s toxicity and potential chronic effects^40^.

These results suggest localized contamination hotspots related to industrial activities and traffic emissions38,39, with heterogeneous spatial distribution influenced by soil properties. Combined with radionuclide activities, soil texture, and organic matter, these findings were used to create a correlation matrix to assess relationships among environmental parameters and potential ecological.

### Correlation matrix between radionuclides, soil texture, organic matter, and heavy metals in soil

**Fig. 32:**
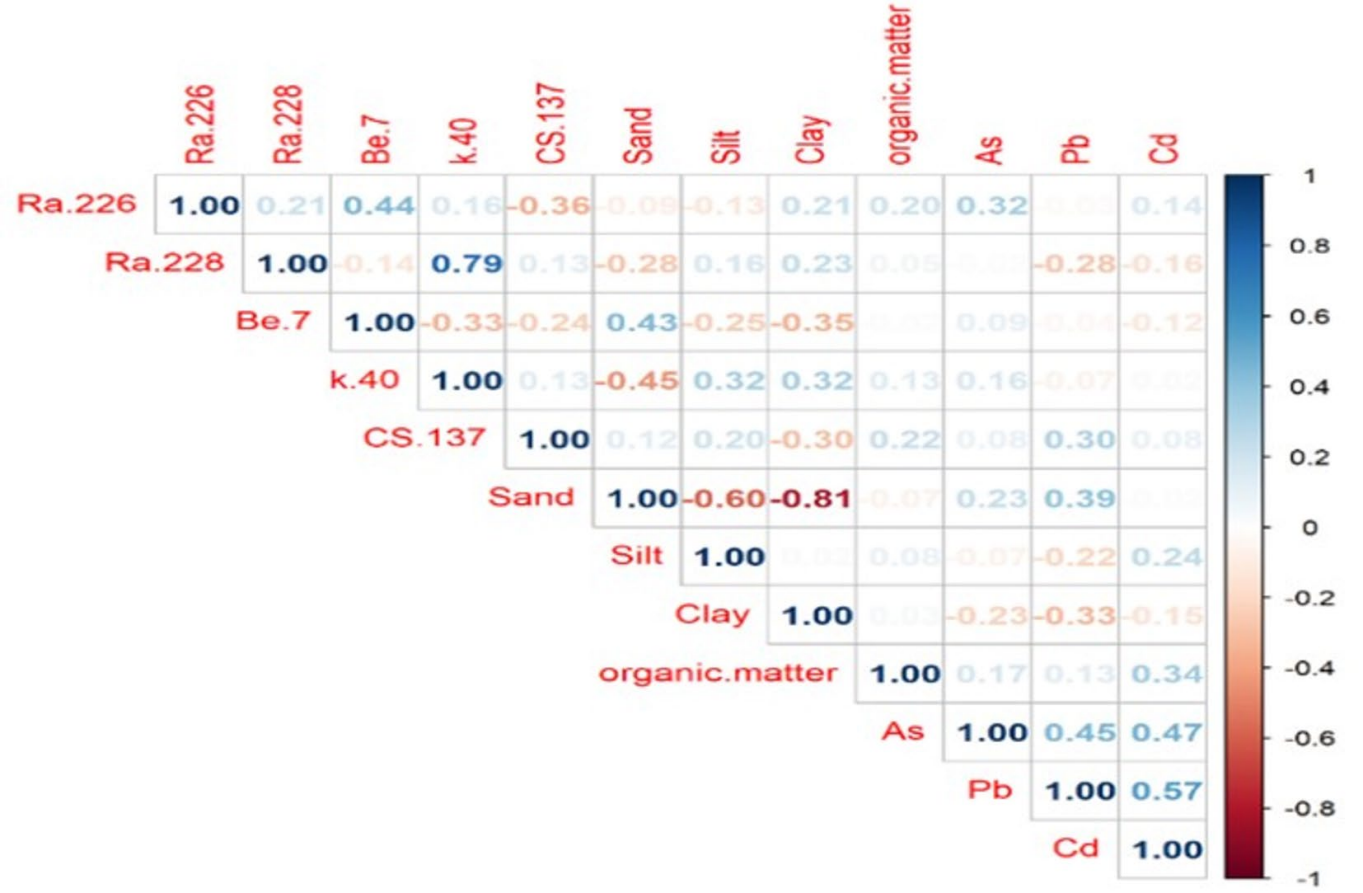
The correlation matrix between radionuclides, soil texture, organic matter, and heavy metals in soil.

Figure 32 displays the correlation matrix that examines the connections among soil organic content, radionuclides, soil texture, and heavy metals.

#### Correlation analysis

The associations between different natural radionuclides (e.g., ^226^Ra, ^228^Ra, ^40^K, ^137^Cs, and^7^B), soil texture components (sand, silt, clay), organic matter, and specific heavy metals (As, Pb, and Cd) were examined using a Pearson correlation matrix, The matrix highlights both strong and weak correlations, with several statistically noteworthy associations, The following is a summary of the correlation matrix’s findings:

Strong positive correlations between Pb and Cd (*r* = 0.57) and between ^228^Ra and^40^K (*r* = 0.79) were found, indicating that these elements have similar geological sources or processes. Conversely, there were significant negative associations between the amount of sand and silt (*r* = − 0.60) and clay (*r* = − 0.81). suggesting that the soil fractions have an inverse relationship. The majority of the variables showed weak to moderate relationships with the radionuclide ^226^Ra, except for an organic matter, which showed minor positive correlations (both *r* = 0.32). Furthermore, a moderate negative correlation (*r* = − 0.35) was found between^7^Be and clay, indicating that soil aggregation and particle size may have an impact on ^7^Be distribution. These results emphasize the intricate relationships among radionuclides, soil properties, and heavy metals, underscoring the effects of both anthropogenic inputs and natural geochemical activity in the region under study^41‚42^.

Most probable sources driving elevated ^226^Ra concentrations:

According to the weight of evidence from our dataset, the observed exceedances of ^226^Ra (33 Bq·kg^−1^ guideline) are most likely caused by a combination of local geogenic and anthropogenic influences. At many affected sites, soluble inputs shown in canal/wastewater and agricultural inputs (phosphate-based fertilizers) are the main contributors, and at locations closest to the plant, power-station-derived TENORM is likely to be a contributing factor. Three lines of data from this study lend credence to this conclusion. A higher ^226^Ra in soils may result from relative uranium enrichment or greater uranium mobility in some areas of the research area, as indicated by the mean Th/U activity ratio (1.96) being lower than the average continental crust value (3.8). Secondly, the correlation matrix (Fig. 32) indicates weak-to-moderate relationships between the suite and ^226^Ra.Third, samples of water and agricultural wastewater taken close to the power plant showed high and spatially patterned ^226^Ra concentrations, with some values surpassing WHO screening levels. This suggests that modern, mobile inputs can transfer radium into soils through surface flow or irrigation.

The most likely primary drivers at agricultural sites (where fertilizer use and irrigation are intensive) are fertilizer-derived inputs and wastewater/irrigation-mediated transport, according to these lines of evidence taken together. For certain sites directly downstream or downwind of the plant, power-station emissions and leachates are probably significant contributors. In certain places, local geology, or background mineralogy, continues to play a role. To quantitatively allocate these sources, we thus advise focused follow-up (mineralogical analysis, sequential leaching, isotopic ratio measurements like ^234^U/^238^U, and geographical overlay with fertilizer application/irrigation maps).

The study’s spatial and correlation patterns show that fertilizer-derived inputs and irrigation-mediated transport are the main sources across the majority of agricultural sites, even though the fossil fuel power plant may locally contribute to soil Ra enrichment through fly ash deposition and leachates. The impact of power-station TENORMs seems to be secondary and site-specific, mostly affecting regions that are directly next to or downstream of the plant.

### Comparison with previous studies

Our results were compared with those of earlier national (Egyptian) and international studies in order to further contextualize the study’s conclusions.

#### Local comparison (Egyptian studies)

Only a small number of studies have evaluated the radiological impacts of industrial and fossil fuel operations in Egypt, but their conclusions are consistent with ours. When Hassan (2023) examined soil radioactivity near Egyptian power plants, he found ^226^Ra levels up to 60 Bq/kg, which is both beyond the international limit of 33 Bq/kg and close to our mean figure of 46.69 Bq/kg. In line with the modest enrichment and low Th/U ratios seen in our samples, Monged et al. (2020) discovered enrichment of ^226^Ra and^40^K in agricultural soils of the Northeastern Nile Valley as a result of fertilizer and irrigation inputs. Ahmed et al. (2021) showed that Egypt’s industrial activity can increase TENORM mobilization, which could account for the higher ^228^Ra seen in our wastewater samples. El-Taher et al. (2019) emphasized the variation in radioactive levels in marine sediments around the Red Sea, demonstrating that radionuclide distribution is greatly influenced by both geology and industrial discharges^29^. When taken as a whole, these regional investigations verify that the increased radioactivity in our research region is indicative of a nationwide trend impacted by both industrial and agricultural operations.

Our results are in line with earlier Egyptian research near power plants and agricultural soils at the national level. Although regional differences are still noticeable, worldwide research provide additional confirmation of these tendencies when viewed in a larger framework.

#### International comparison

Similar findings have been seen near fossil fuel and industrial sites worldwide. Wide variations in soil-to-plant transfer factors (TFs) of ^226^Ra, ^40^K, and ^137^Cs were noted by Saenboonruang et al. (2018) in Thailand and Van et al. (2020) in Vietnam. These findings are in line with the interspecific variations we found, especially the elevated ^226^Ra in Triticum aestivum and the high^40^K uptake in *Artemisia sp*. In Nigeria Waida et al.^42^ found that soil and plant activity of ^226^Ra and ^228^Ra were higher than our results, indicating that West Africa has more robust geological contributions. These results corroborate our conclusion that localized enrichment is noticeable and mainly associated with fossil fuel emissions and agricultural practices, even while activity levels close to the West Delta Power Station stay within the global background range.

### Study limitations and future recommendations

This study provides valuable baseline data on radiological and radioecological risks surrounding the West Delta fossil-fuel power station; however, several limitations should be acknowledged. First, the sampling campaign was conducted during a single season and included a limited number of representative sites. As a result, potential temporal variations in radionuclide concentrations due to seasonal or hydrological changes could not be fully assessed. Second, only gamma-emitting radionuclides were quantified in this study, while alpha- and beta-emitting radionuclides such as uranium and strontium were not measured directly. Third, the assessment was based on external exposure pathways and soil-to-plant transfer; other possible exposure routes, including inhalation and ingestion via food chains, were not evaluated. In addition, the interpretation of radiological hazard indices was based on international models that may not fully capture site-specific factors such as local diet, soil composition, or meteorological conditions.

Future studies should therefore aim to extend the spatial and temporal coverage, include multiple sampling campaigns across different seasons, and incorporate a wider range of radionuclides and exposure pathways. Integration of geochemical modeling and biomonitoring data is also recommended to improve understanding of radionuclide mobility, bioavailability, and long-term ecological impact. These efforts will enhance the robustness of radiological risk assessments and support evidence-based regulatory and environmental management strategies in Egypt.

## Conclusion

This study provides the first comprehensive radiological and radioecological assessment of the environment surrounding the West Delta fossil-fuel power station in Egypt. The measured radionuclide concentrations in soil indicated moderate enrichment of ^226^Ra and^40^K, while ^137^Cs remained within natural background levels. Although most radiological indices (Ra__eq_, H__ex_, H__in_, AED, ELCR, and ELGR) were within internationally accepted limits, water and wastewater samples exhibited ^226^Ra and ^228^Ra levels exceeding WHO screening reference values, suggesting potential localized impacts that warrant continued evaluation.

Plant uptake patterns revealed that *Triticum aestivum* and *Ricinus communis* showed higher transfer factors, supporting their suitability as bioindicator species, while other species demonstrated variable accumulation behavior. The spatial distribution of heavy metals particularly Pb and Cd along with variations in soil structure and organic matter content, indicates combined agricultural and industrial influences on the study area.

Overall, the findings establish an important environmental baseline and highlight the need for regular monitoring to track potential future accumulation trends and assess long-term ecological and public exposure risks. However, the current study is limited by the temporal sampling window and spatial coverage, and future work incorporating seasonal variability, larger sampling density, and isotopic source apportionment would strengthen understanding of contaminant pathways and potential health implications.

## Data Availability

The datasets used and/or analysed during the current study available from the corresponding author on reasonable request.

## Abbreviations

^238^U: Uranium-238
^235^U: Uranium-235
^232^Th: Thorium-232
^40^K: Potassium-40
^137^Cs: Cesium-137
^226^Ra: Radium-226
^228^Ra: Radium-228 (representative of the Th-232 decay series)
^214^Pb: Lead-214
Ra_eq_: Radium equivalent
H_ex_: External hazard index
H_in_: Internal hazard index
D: Absorbed dose rate in air
AED: Annual effective dose rate
ELCR: Excess lifetime cancer risk
ICP-OES: Inductively coupled plasma-optical emission spectroscopy
HPGe: High-purity germanium detector
EPA: Environmental Protection Agency
IAEA: International Atomic Energy Agency
ICRP: International commission on radiological protection
NORMs: Naturally occurring radioactive materials
UNSCEAR: United nation scientific committee on the effects of atomic radiation
WHO: World Health Organization
